# Overcoming the Challenge of Confident Identification Among Two Related Groups of 17‐Methyl Steroids by GC–MS

**DOI:** 10.1002/jssc.70334

**Published:** 2025-12-18

**Authors:** Jakob Steff, Xavier de la Torre, Francesco Botrè, Maria Kristina Parr

**Affiliations:** ^1^ Institute of Pharmacy Freie Universität Berlin Berlin Germany; ^2^ Laboratorio Antidoping FMSI Largo Giulio Onesti 1 Rome Italy; ^3^ Department of Chemistry “Ugo Schiff” University of Florence Sesto Fiorentino Italy

**Keywords:** anabolic androgenic steroids, anti‐doping analysis, chromatographic separation, mass spectrometry, method development

## Abstract

Tetrahydromethyltestosterone (THMT) and 20‐hydroxymethyl‐18‐nortetrahydromethyltestosterone (20OHnorTHMT) are metabolites of the anabolic androgenic steroids methyltestosterone and metandienone. Both molecular structures are used as markers in anti‐doping analysis. There are eight reasonable diastereomeric structures of each group relevant for metabolic purposes. Highly sophisticated mass spectrometers fail to confidently differentiate these closely related, yet non‐isomeric and non‐isobaric groups of molecules. Due to the low abundance of the molecular ion, high‐resolution mass spectrometry provides shared fragment ions that challenge identification by extracted ion chromatograms out of full scan mode acquisitions. Further on, tandem mass spectrometry uses partly the same ion transitions for both groups of targeted analytes. Thus, a reliable chromatographic separation is absolutely necessary. Therefore, a gas chromatographic method using a DB‐5 ms capillary column (30 m, 0.25 mm, and 0.25 µm) was developed. Hence, discrimination between the two groups was enabled, and a confident structural assignment among the eight diastereomers was achieved. This case study contributes to a higher quality of anti‐doping analysis, but even further raises awareness of the importance of chromatographic separation in cases of insufficient mass spectrometric discrimination.

## 1 Introduction

Analytical methods and possibilities in the field of bioanalytics, such as metabolomics, toxicology, or anti‐doping analysis, have improved remarkably over the last decades. For instance, more diverse separation techniques like ion mobility spectrometry–mass spectrometry (IMS–MS) or supercritical fluid chromatography (SFC) are gaining in importance in these disciplines [1, 2, 3, 4]. Especially the identification of analytes using mass spectrometry systems shows highly increased sensitivity, selectivity, resolution, and mass range, among others, by constant instrumental progress [5, 6, 7]. For example, higher scan rates of mass spectrometers enable the analysis of hundreds of compounds in a single run [8].

Despite these advancing developments in mass spectrometry, the approach of hyphenating mass spectrometry to chromatography still faces difficulties if it comes to distinguishing and annotating isomers. This limitation occurs in different disciplines.

Among others, in the subject of lipidomics, the structural similarities of lipids and fatty acids challenge chromatography and mass spectrometry equally [9]. Mostly, if not coupled to additional techniques, a full characterization of lipidomic samples cannot be achieved [10, 11].

An even bigger challenge for analytical chemistry poses the field of new psychoactive substances (NPS) [12]. Highly similar molecular structures and regioisomers often provide the same high‐resolution mass spectrometry (HRMS) data and can also show identical transitions in tandem mass spectrometry (MS/MS) experiments [13]. A combination of different analytical techniques is also commonly required for a confident structural identification of NPS [14].

Overall, it becomes even more challenging when it is aimed for differentiation of diastereomers. For instance, the endogenous androgenic steroids androsterone, epiandrosterone, etiocholanolone, and epietiocholanolone exhibit the same molecular ion, the same fragments in MS spectra, only differing in the relative intensities among each other [15, 16]. Therefore, a sufficient chromatographic separation by LC or gas chromatography (GC) is required for proper identification. Similar problems arise during the analysis of enantiomers, for which mass spectrometry is considered blind. Only by the introduction of a diastereomeric environment by derivatization or complexation, a differentiation of enantiomers is partially achievable by mass spectrometry [17, 18].

These challenges are very well known and described by the scientific community. In general, the more impressive current analytical achievements become, the more important it is to constantly scrutinize achievements for sufficient improvement.

With this work, we would like to raise awareness for a supposedly simple potential pitfall in analytical chemistry and how to avoid it—common fragment ions/ion transitions and no chromatographic separation among similar, but non‐isomeric molecule structures.

**FIGURE 1:**
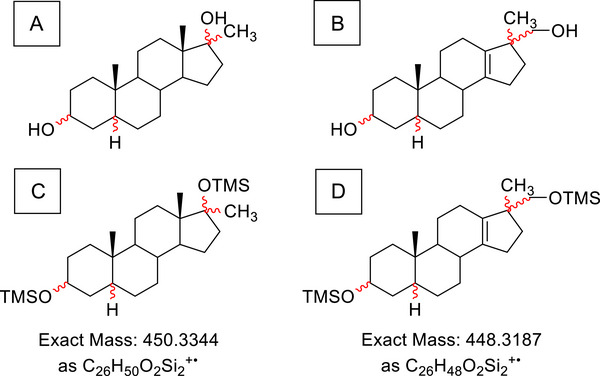
Chemical structures of (A) THMT, (B) 20OHnorTHMT, (C) THMT as bis‐TMS derivative, and (D) 20OHnorTHMT as bis‐TMS derivative. Relevant stereocenters are indicated in red wavy bonds.

In anti‐doping research and general steroid metabolism, tetrahydromethyltestosterone (THMT, **T**) and 20‐hydroxymethyl‐18‐nortetrahydromethyltestosterone (20OHnorTHMT, **N**) represent two classes of metabolite structures of the anabolic androgenic steroids metandienone (MD) and methyltestosterone (MT) [19, 20]. THMT exhibits eight and 20OHnorTHMT six stereocenters, but in both groups, only positions 3, 5, and 17 (Figure 1) are of relevance according to the current state of knowledge regarding metabolic alterations. This leads to eight diastereomeric structures in each case, which are depicted in Table 1. So far, **T1**, **T3**, **T7**, **N1,** and **N3** have been detected after administration in humans [19]. These 16 molecule structures pose a significant challenge for routine analytical approaches and visualize the risk of a false annotation of signals and, thereby, an insufficient identification.

**TABLE 1:** Assignment of stereochemistry of the considered diastereomers.

	5α	5β	
**3α**	T1/N5	T3/N7	**17α‐CH_3_**
**3β**	T2/N6	T4/N8	
**3α**	T5/N1	T7/N3	**17β‐CH_3_**
**3β**	T6/N2	T8/N4	

## 2 Materials and Methods

### 2.1 Chemicals

17α‐Methyl‐5α‐androstane‐3α,17β‐diol (**T1**), 17α‐methyl‐5α‐androstane‐3β,17β‐diol (**T2**), 17α‐methyl‐5β‐androstane‐3α,17β‐diol (**T3**), 17α‐methyl‐5β‐androstane‐3β,17β‐diol (**T4**), 17β‐methyl‐5α‐androstane‐3α,17α‐diol (**T5**), 17β‐methyl‐5α‐androstane‐3β,17α‐diol (**T6**), 17β‐methyl‐5β‐androstane‐3α,17α‐diol (**T7**), and 17β‐methyl‐5β‐androstane‐3β,17α‐diol (**T8**) have been synthesized in‐house [21]. The synthesis of 17α‐hydroxymethyl‐17β‐methyl‐18‐nor‐5α‐androst‐13‐en‐3α‐ol (**N1**), 17α‐hydroxymethyl‐17β‐methyl‐18‐nor‐5α‐androst‐13‐en‐3β‐ol (**N2**), 17α‐hydroxymethyl‐17β‐methyl‐18‐nor‐5β‐androst‐13‐en‐3α‐ol (**N3**), 17α‐hydroxymethyl‐17β‐methyl‐18‐nor‐5β‐androst‐13‐en‐3β‐ol (**N4**), 17β‐hydroxymethyl‐17α‐methyl‐18‐nor‐5α‐androst‐13‐en‐3α‐ol (**N5**), 17β‐hydroxymethyl‐17α‐methyl‐18‐nor‐5α‐androst‐13‐en‐3β‐ol (**N6**), 17β‐hydroxymethyl‐17α‐methyl‐18‐nor‐5β‐androst‐13‐en‐3α‐ol (**N7**), and 17β‐hydroxymethyl‐17α‐methyl‐18‐nor‐5β‐androst‐13‐en‐3β‐ol (**N8**) was accomplished in‐house as well [22]. Ammonium iodide (≥ 99%) and ethanethiol (97%) were purchased from Sigma‐Aldrich GmbH (Taufkirchen, Germany). *N*‐Methyl‐*N*‐(trimethylsilyl)trifluoroacetamide (MSTFA) was obtained from Chemische Fabrik Karl Bucher GmbH (Waldstetten, Germany).

### 2.2 Derivatization Prior to GC–MS Analysis

Compounds were converted to their bis‐TMS derivatives as depicted in Figure 1C,D. Therefore, 0.5 µg of dried compounds was treated with 100 µL TMIS reagent (MSTFA, ammonium iodide, and ethanethiol, 1000:2:6, v:w:v) and incubated at 70°C for 20 min.

### 2.3 GC‐QqQ‐MS With Temperature Program GC 1

**FIGURE 2:**
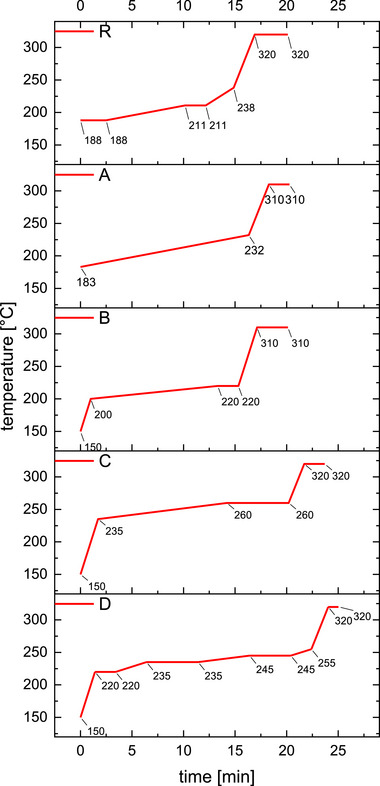
Temperature programs of GC methods (R: previously reported method [19]; A–D: presented chromatograms depicted in Figure 4).

Initial MS/MS experiments with the routine method for anti‐doping analysis [19] were performed on an Agilent 7890A GC system coupled to an Agilent 7010 triple quadrupole mass spectrometer. It was equipped with an Agilent HP1 column (17 m, 0.20 mm, 0.11 µm) and helium as carrier gas with a constant flow of 0.84 mL/min. The oven program was applied as indicated in Figure 2R: 188°C, hold for 2.5 min, +3°C/min to 211°C, hold for 2.0 min, +10°C/min to 238°C, +40°C/min to 320°C, hold for 3.2 min. The injection was performed with 2 µL, a split ratio of 20:1, and an inlet temperature of 280°C. Ion source temperature was set to 280°C, an ionization energy of 70 eV was applied, and ion transitions of the integrated targeted compounds are depicted in Table 2.

**TABLE 2:** Routine conditions in anti‐doping analysis targeting T1, T2, T3, N1, and N3 (RT = retention time and detection window ±0.4 min).

Compound	RT (min)	Ion transitions (*m/z) and* collision energies (eV)
17α‐Hydroxymethyl‐17β‐methyl‐18‐nor5β‐androst‐13‐en‐3β‐ol **(N3)**	9.80	345.0 → 255.0 (5) 345.0 → 173.0 (20) 345.0 → 159.0 (10)
17α‐Hydroxymethyl‐17β‐methyl‐18‐nor5β‐androst‐13‐en‐3β‐ol (**N1**)	10.13	345.0 → 255.0 (5) 345.0 → 173.0 (20) 345.0 → 159.0 (10)
17α‐Methyl‐5α‐androstane‐3α,17β‐diol (**T1**)	13.23	450.4 → 365.0 (10) 450.4 → 345.0 (10) 450.0 → 261.0 (10) 318.0 → 199.0 (10) 318.0 → 187.0 (10) 318.0 → 182.0 (10)
17α‐Methyl‐5β‐androstane‐3α,17β‐diol (**T3**)	13.34	270.0 → 199.0 (30) 270.0 → 171.0 (30) 270.0 → 157.0 (30) 228.0 → 174.0 (5)
17α‐Methyl‐5α‐androstane‐3β,17β‐diol (**T2**)	14.80	435.4 → 213.0 (20) 435.4 → 199.0 (20) 360.0 → 255.0 (30) 360.0 → 213.0 (20)

### 2.4 GC–MS With Temperature Program GC 2

Method development of the chromatographic method was performed on a 7890 gas chromatograph (Agilent Technologies Inc., Santa Clara, CA, USA) coupled to a 5975C single quadrupole mass‐selective detector (Agilent Technologies Inc.) with helium as carrier gas, with a constant flow rate of 1 mL/min. The inlet temperature was set to 300°C, the injection volume was 2 µL, and the split ratio 16:1. The ion source temperature was set to 230°C, and an ionization energy of 70 eV was applied, and full scan mode ranged from *m/z* 40 to 1000. As starting point for method development, an established method [23, 24, 25, 26] equipped with an Agilent HP1 column (17 m, 0.20 mm, 0.11 µm) and the following temperature program was used: 183°C, +3°C/min to 232°C, +40°C/min to 310°C, hold for 2 min.

Within method development, several temperature programs were tested. Details are stated in Supporting Information.

### 2.5 GC‐QTOF‐MS With Temperature Program GC 3

GC‐EI‐QTOF‐MS experiments were performed on a 7890B/7250 (Agilent Technologies Inc., Milano, Italy) equipped with an Agilent DB‐5 ms fused silica capillary column (30 m, 0.25 mm, 0.25 µm, (5% phenyl)‐methylpolysiloxane). Parameters for the acquisition were applied as gathered in Table 3. Mass calibration was performed twice at the beginning and repeated after every second injection of a sample.

**TABLE 3:** Applied parameters for GC‐QTOF‐MS experiments.

Acquisition parameters		Oven temperature program	
Carrier gas	Helium	Time (min)	Temperature (°C)
Injection volume	1 µL	0	150
Inlet temperature	300°C	1.4	220
Split ratio	15:1	3.4	220
Constant flow	1 mL/min	6.4	235
Full scan mode	*m/z* 50–850	114	235
Ionization energy	15 eV	16.4	245
Source temperature	230°C	20.4	245
		22.4	255
		24.025	320
		26.025	320

### 2.6 Data Analysis

Data evaluation was performed using Agilent MassHunter Qualitative Analysis 10.0 (Agilent Technologies, Santa Clara, CA, USA). The chromatographic resolution (*R*) of peak pairs was also determined using Agilent MassHunter Qualitative Analysis 10.0 based on the European Pharmacopoeia according to the following formula:

$$R=\frac{1.18\;\times \left(t_{R,2}-\;t_{R,1}\right)}{W_{50,1}+W_{50,2}}$$

(*t_R_*_,1/2_: retention times of peaks; W_50,1/2_: peak width at 50% of the height of the peak)

## 3 Results

### 3.1 Comparison of Analyte Separation by Routinely Used GC–MS Temperature Programs

**FIGURE 3:**
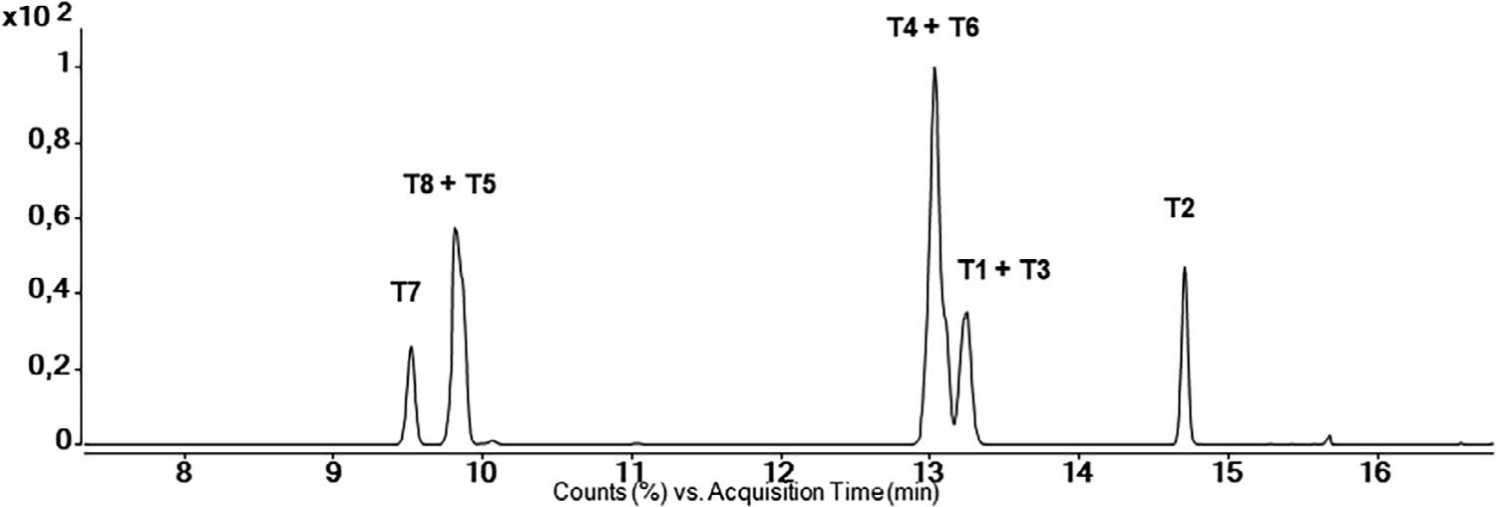
GC‐QqQ‐MS (2.3) total ion chromatogram of a mixture of **T1**–**T8**.

**FIGURE 4:**
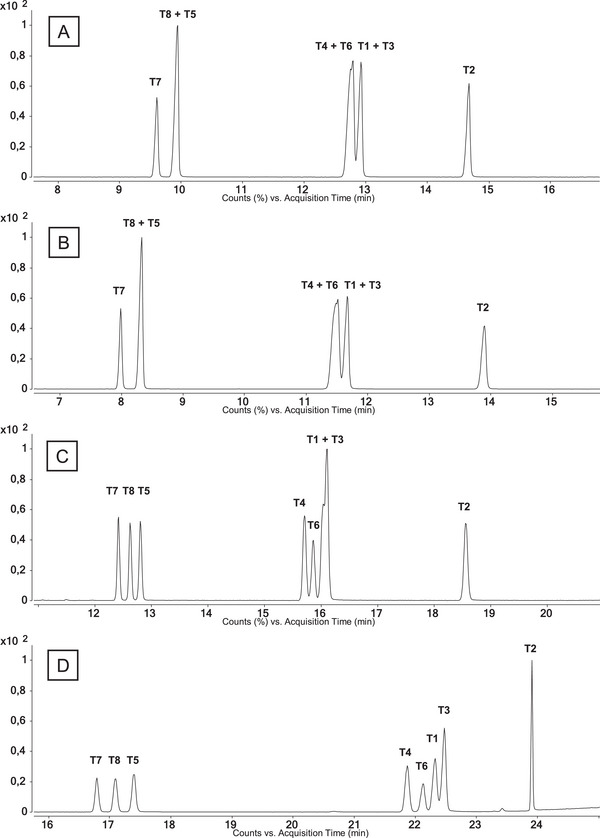
GC–MS (2.4) chromatograms from method development on a HP1 column (A, B) and a DB‐5 ms column (C, D) with different temperature programs depicted in Figure 2 and described in the Supporting Information.

Initially, the conditions of the temperature program GC 1 were tested by GC‐QqQ‐MS (2.3, Figure 2R) as routinely applied in anti‐doping analysis from a standard method for steroids [27] using an Agilent HP1 column (17 m, 0.2 mm, 0.11 µm, 100% dimethylpolysiloxane). A mixture of all eight THMT isomers was analyzed. The resulting separation is depicted in Figure 3. In contrast to the latter method (Figure 2R), the only slightly different and well‐established temperature program GC 2 (Figure 2A) [23] using the same HP‐1 column as well was tested with the mixture of THMT isomers. The main difference can be found in the heat program with one constant heating rate at the beginning of the method. As illustrated in Figure 3 and Figure 4A, in both approaches, only two out of the eight isomers (**T7** + **T2**) are eluted without co‐elution with another diastereomer.

### 3.2 Gas Chromatography Method Development

Details of the temperature programs presented in the following can be found in Supporting Information. The method routinely used, applying temperature program GC 2 (2.4, Figure 4A), was chosen as the starting point for the method development targeting the separation of all the above‐mentioned isomers of the analytes. It was performed on a GC‐SQ‐MS (2.4). An adjustment of the temperature program (Figure 2B), starting at 150°C with a short and steep ramp (50°C/min for 1 min) instead of 183°C and a lower heating rate (3°C/min to 1.5°C/min), did not impact the separation of the critical pair. It only led to an earlier elution of about 1 min for the first eluting peak. Whereas the exchange of the column to an Agilent DB‐5 ms (30 m, 0.25 mm, 0.25 µm, (5% phenyl)‐methylpolysiloxane) and a general elevation of the temperatures in the method including a longer hold time than before (Figure 2B,C) resulted in an improved separation. All isomers except **T1** and **T3** showed separate elution with a resolution of at least 1.5.

Finally, adding longer hold times in‐between sections of increased heating and therefore prolonging the method resulted in the temperature program GC 3 (Figure 2D). Thereby, a separation of all eight isomers was accomplished (Figure 4D). Initially co‐eluting **T8** and **T5** are now showing a resolution of *R*_T8,T5_ = 2.5. Furthermore, **T4**, **T6**, **T1,** and **T3,** which nearly co‐eluted completely in the initial method (Figure 4A), finally exhibited sufficient separation (*R*_T4,T6_ = 1.9; *R*_T6,T1_ = 1.5; *R*_T1,T3_ = 1.2). Separation of the complete set of THMTs and 20OHnorTHMTs applying the temperature program GC 3 and resulted in the following elution order: **T7** < **N3** < **T8** < **N4** < **T5** < **N1** < **N7** < **N5** < **N2** < **T4** < **T6** < **T1** < **T3** < **N6** < **T2** (Figure 7). Resulting retention times after transfer to the GC‐QTOF‐MS system (2.5, Table 3) are listed in Table 4. For the latter, the retention time of **N8** cannot be stated, due to the nonavailability after purification during the synthetic process. It must be noted that the GC–MS system used during method development exhibits longer retention times of **T1**–**T8** (Figure 4D) than on the GC‐QTOF‐MS system, as reported in Table 4. Inter‐instrumental differences in the total system setup are considered an explanation for the difference in retention time. Nevertheless, the resolution of the critical pairs was only affected minimally: *R*_T8,T5_ = 2.3; *R*_T4,T6_ = 1.8; *R*_T6,T1_ = 1.3; *R*_T1,T3_ = 1.2.

**TABLE 4:** Analytes (A) and their retention times of the GC–MS routine method with the HP1 column (GC 1, 2.3, Figure 2R) and the routinely applied GC–MS method with the HP1 column (GC 2, 2.4, Figure 2A), and the developed GC–MS method with the DB‐5 ms column (GC 3, 2.5, Figure 2D) (n.d. = not determined in preliminary experiments).

A	GC 1 HP1 RT (min)	GC 2 HP1 RT (min)	GC 3 DB‐5 ms RT (min)
**T7**	9.53	9.60	14.01
**N3**	9.80	9.85	14.14
**T8**	9.82	9.94	14.25
**N4**	9.99	10.04	14.35
**T5**	9.82	9.94	14.53
**N1**	10.07	10.16	14.64
**N7**	n.d.	n.d.	15.43
**N5**	n.d.	n.d.	15.89
**N2**	n.d.	12.52	18.10
**T4**	13.03	12.79	18.23
**T6**	13.03	12.79	18.50
**T1**	13.23	12.92	18.69
**T3**	13.23	12.92	18.87
**N6**	n.d.	n.d.	19.04
**T2**	14.71	14.67	21.83

### 3.3 Assessment and Application of Mass Spectrometric Characteristics of THMT and 20OHnorTHMT by GC‐QTOF‐MS

**FIGURE 5:**
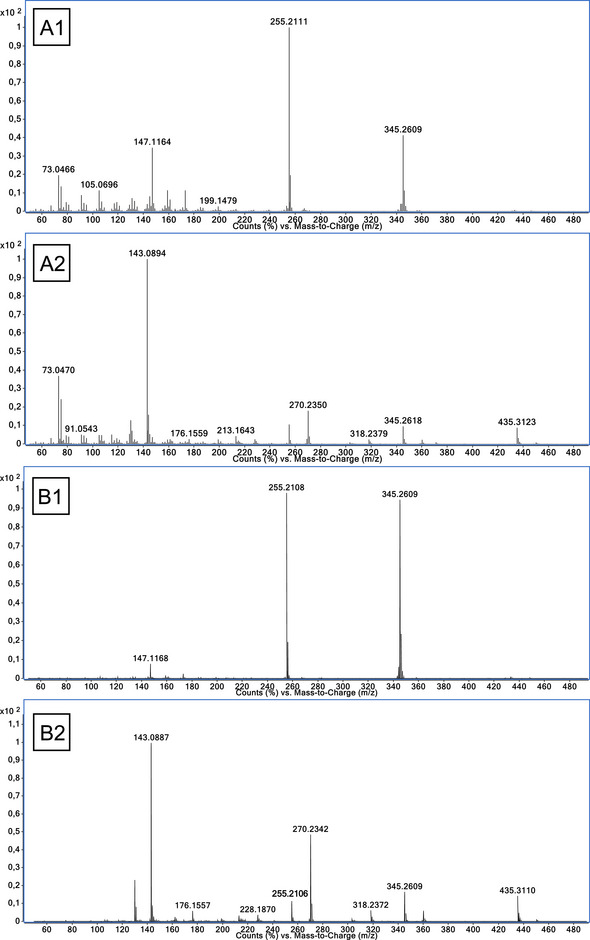
Comparison of HRMS spectra of N4 and T4 as bis‐TMS derivatives with different ionization energies (A1: **N4**, 70 eV; A2: **T4**, 70 eV; B1: **N4**, 15 eV; B2: **T4**, 15 eV).

GC‐QTOF‐MS measurements of the 20OHnorTHMT diastereomers with standard ionization energy of 70 eV yielded mass spectra exhibiting rather poor diversity of formed fragment ions (exemplary illustrated by **N4** in Figure 5A1). In all eight 20OHnorTHMT isomers, the fragment ions *m/z* 345 ([M^+•^‐^•^CH_2_OTMS]) and *m/z* 255 ([M^+•^‐^•^CH_2_OTMS‐TMSOH]) are by far the most abundant, followed by *m/z* 147 (accurate mass = 147.1168). In TMS derivatized samples, *m/z* 147 can be explained by the fragment [Si(CH_3_)_3_‐O‐Si(CH_3_)_2_]^+^ (exact mass = 147.0656), which seems rather unlikely due to the spatial distance of the two TMS groups in the molecule and is supported by the significant mass difference [28]. Instead, the composition of this fragment may be explained by [C_5_H_11_]^+•^ (exact mass = 147.1168) exhibiting a mass error to the observed *m/z* 147.1174 of −0.18 ppm. Furthermore, only low‐abundant indications of the molecular ion M^+•^ = *m/z* 448 or the fragment [M^+•^‐15] = *m/z* 433 are observable.

On the other hand, the fragmentation of the THMT isomers leads to a higher number of fragment ions as extensively described by our group before [16]. Generally, the fragment ion *m/z* 143 represents the base peak with a high intensity in comparison to the remaining fragments, as it can be seen for **T4** in Figure 5A2. Noticeably, *m/z* 345 and *m/z *255 also derive from THMT, and the molecular ion M^+•^ = *m/z* 450 is barely detectable.

Thus, to increase the abundance of M^+•^ or high mass fragments like [M^+•^‐15] of THMT and 20OHnorTHMT, experiments with low energy ionization (LEI) MS were conducted. The results of these measurements of **N4** and **T4** with 15 eV ionization energy are exemplary depicted in Figure 5B1,B2. Still, it can be seen that the molecular ion was neither sufficiently detectable for **N4** (exact mass M^+•^ = 448.3187) nor for **T4** (exact mass M^+•^ = 450.3344). Furthermore, a comparison of the spectra with either 15 eV or 70 eV ionization energy (Figure 5A,B) shows for 20OHnorTHMT a further decrease of the variety of detectable fragment ions, but a further increase of the abundance of the prominent fragments *m/z* 345 and *m/z* 255. On the other hand, in THMT, a decrease of the abundance of the base peak *m/z* 143 and low mass fragment ions was observed going hand in hand with higher intensities of the characteristic high mass fragments (*m/z* 435, *m/z* 360, *m/z* 345, *m/z* 318, *m/z* 270, and *m/z* 255) [16].

**FIGURE 6:**
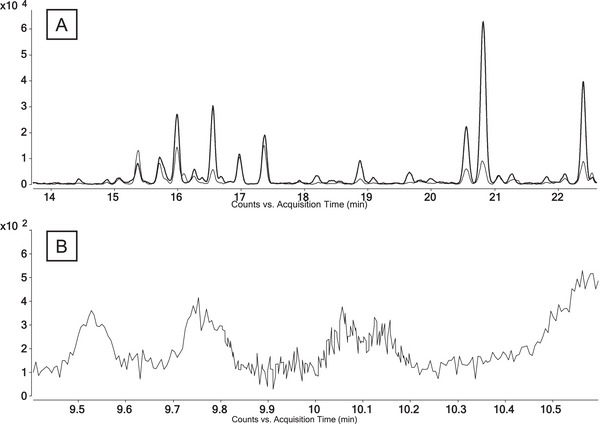
Chromatograms of human blank urines of male volunteers (A) applying GC 3 method on a GC‐QTOF‐MS (2.5, EIC [mass extraction window of ±10 ppm] *m/z* 345.2609 [continuous line] and *m/z* 255.2109 [dotted line]) and (B) applying GC 1 method on a GC‐QqQ‐MS (2.3, MRM, *m/z* 345 → 255).

Following, human blank urine was prepared as routinely performed in anti‐doping analysis, including a liquid–liquid extraction and TMS derivatization as reported by Steff et al. [22]. Measurements were performed as described in 2.3 and 2.5. Figure 6 shows the extracted ion chromatograms (EIC) of *m/z* 345.2609 and *m/z* 255.2109 of the injection to the GC‐QTOF‐MS system applying temperature program GC 3, indicating an additional elution of several matrix compounds during the time of elution of the targeted analytes (Table 4).

**FIGURE 7:**
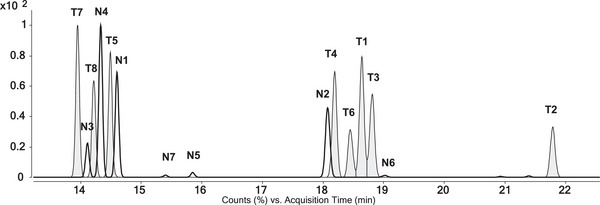
EICs (*m/z* 255.2109) of a mixture of 20OHnorTHMT (transparent) and of THMT (gray) applying the final optimized GC‐QTOF‐MS method (2.5).

## 4 Discussion

### 4.1 Chromatographic Separation of THMT and 20OHnorTHMT

Concerning the low selectivity of mass spectrometry for these analytes (as further described in 4.2), a confident identification can only be performed by a sufficient chromatographic separation prior to MS detection.

As described in 4.2.3, ESI–MS is not a promising strategy. Hence, techniques dependent on ESI–MS, such as LC‐ESI‐MS, SFC‐ESI‐MS, and IMS–MS, are not suitable options for the bioanalysis of THMT and 20OHnorTHMT. Consequently, only GC poses a practical chromatographic system in the case of THMT and 20OHnorTHMT.

Therefore, converting steroids into their TMS‐derivatives was chosen, because it has been proven useful in increasing gas chromatographic performance by formation of more volatile and thermally stable derivatives [29, 30]. However, Loke et al. have shown that the application of a routinely used method (temperature program GC 1 and conditions of 2.3, Figure 2R) for GC–MS for anti‐doping purposes does not provide sufficient chromatographic performance to confidently discriminate all potential analytes without further analysis, due to co‐elution of **T5** and **N3** (described in 4.2.1) [19].

In addition to the temperature program GC 1 (Figure 2R), another established method with temperature program GC 2 (2.4) [23, 24, 25, 26] was tested, but also did not ensure sufficient separation of, in this case, eight THMT isomers, as depicted in Figure 4A.

Therefore, a method with a temperature program GC 3 was developed. Significantly better performance was finally achieved by adjusting the analytical parameters. One part was the prolongation of the heating program by inserting temperature plateaus. Thus, by the increase of the elution time, a longer interaction of these very similar structures was granted so that little structural differences could show their effect. In parallel, the stationary phase was changed from 100% dimethylpolysiloxane to (5% phenyl)‐methylpolysiloxane. Even though the chemical composition is different in the new phase bearing phenyl groups in the polymer backbone, both phases are considered rather unpolar [31]. Most likely, the increase in the column length from 17 to 30 m played a major role. On the other hand, it was reported by Massé et al. that several chromatographic approaches using both latter stationary phases with a length of 30 m did not manage to separate the peaks of **T1** and **T3** [32]. Hence, the key to separation is the interplay of column length and heat program. The above‐mentioned routine anti‐doping method (temperature program GC 1, Figure 2R) separates **T1** and **T3** with a retention time difference of ΔRT = 0.14 min in accordance with Loke et al. [19]. The final method with temperature program GC 3 (2.5, Figure 2D) increases the separation to ΔRT = 0.18 min. The final separation of all analytes leads to the necessary confidence in identification at the cost of a prolongation of the total run time to 26 min.

### 4.2 Limitations of Mass Spectrometry

The following discussion depicts that in this case, highly sophisticated mass spectrometry systems (tandem mass spectrometry and HRMS) are unable to confidently assign diastereomers and closely related non‐isobaric molecule structures— THMT and 20OHnorTHMT. Solely, chromatographic separation using the method with temperature program GC 3 (2.5, Figure 2D) enabled an unequivocal identification of latter structures (4.1).

#### 4.2.1 Tandem Mass Spectrometry

Tandem mass spectrometry was found unable to reliably differentiate between **T** and **N**. Due to the poor diversity of formed fragment ions of 20OHnorTHMT (Figure 5), there are limited options for choosing robust ion transitions with sufficient intensities for GC‐QqQ‐MS analysis. Ion transitions *m/z* 345.0 → 255.0, *m/z* 345.3 → 173.0, and *m/z* 345.3 → 159.0 are commonly chosen for 20OHnorTHMT [19, 22]. Despite the expectation toward MS/MS to be extremely selective, it must be reported that the use of these ion transitions affects the reliability of diastereomeric assignment. It was shown that these conditions do not only cover 20OHnorTHMT but also THMT. Loke et al. demonstrated that *m/z* 345.3 → 173.0 also includes **T5** and **T7**. Furthermore, **T5** elutes closely to **N3,** preventing confident assignment of the signal if the temperature program GC 1 and conditions of 2.3 are applied [19]. In this case, it is only possible to distinguish between THMT and 20OHnorTHMT by adding a selective ion transition for THMT *m/z* 450 → 345. However, not all THMTs provide sufficient abundance of the molecular ion [M]^•+^ = *m/z* 450 (Figure 5A2,B2), which makes this approach not all‐encompassing [16] (further elaboration in 4.2.2). Furthermore, it was observed in an excretion study that some individuals have also shown co‐elution regarding the applied transitions (*m/z* 345.0 → 255.0, *m/z* 345.3 → 173.0, *m/z* 345.3 → 159.0) and conditions of 2.3 with temperature program GC 1. So far, unidentified components of the matrix are hampering a confident identification of **N3** for long‐term detection [22]. This is further supported by Figure 6B depicting a chromatogram of the transition *m/z* 345.0 → 255.0 of a blank urine sample. It shows an eluting signal at RT 9.75 min, which is in close proximity to **N3** eluting at RT 9.8 min.

Nevertheless, high selectivity and sensitivity are of importance for these substances, because of their very low concentrations in post‐administration urine samples. Therefore, the analysis of these substances in biological specimens required the use of MS/MS systems, but only chromatographic separation enables their confident assignments.

#### 4.2.2 High‐Resolution Mass Spectrometry‐

HRMS experiments by GC‐QTOF‐MS can confirm the molecular composition of analytes (Figure 1; **T**: C_26_H_50_O_2_Si_2_; **N**: C_26_H_48_O_2_Si_2_). It might sound simple, but to do so, the molecular ion must be detectable. Due to the extensive fragmentation of THMT and 20OHnorTHMT using EI–MS, most of the diastereomers show no molecular ion [16]. Also, the application of LEI (15 eV instead of typically 70 eV) makes no significant difference, as it is depicted in Figure 5. Therefore, already confident differentiation between the two groups is hampered. Further, identification by comparing mass spectra among the two groups without considering the molecular ion is challenging, because the most abundant fragment ions of 20OHnorTHMT, *m/z* 255 and *m/z* 345 (Figure 5), are also exhibited by THMT, and also in several endogenous matrix components [8]. This is further illustrated by signals of the EICs (*m/z* 345.2609 and *m/z* 255.2109) of mere blank urine, indicating the co‐elution of endogenous matrix components in Figure 6.

Differentiation among the eight diastereomers of both THMT and 20OHnorTHMT can be managed theoretically by an extensive understanding of the relative intensities and their respective ratios of characteristic fragment ions. But this approach also reaches its limitations regarding inter‐measurement variations of the relative abundance in (GC–)MS experiments [33, 34, 35] and the above‐mentioned influence of matrix components influencing the ratios of relative intensities.

Finally, HRMS can only be used for the analysis of these analytes by finding robust fragment ions with adequate intensities, which seems to be challenging. Anyway, a chromatographic separation for this approach is still deeply needed.

#### 4.2.3 Electron Ionization Mass Spectrometry

The obstacles of low selectivity, especially for 20OHnorTHMT in mass spectrometry, described above, are mainly due to the extensive fragmentation when using EI–MS despite applying LEI. In the case of 20OHnorTHMT, as in Figure 5, LEI only led to a reduction of low mass fragment ion abundances and therefore decreased the structural information deducible from the spectra. A so‐called cold electron ionization (CEI) might deliver higher abundances of the molecular ion instead, but this brings high instrumental requirements, which are not implemented in most laboratories [36, 37]. In theory, this problem can be solved by switching from hard ionization to soft ionization. Thus, the application of chemical ionization (CI), as it is considered a soft ionization technique, often provides more abundant molecular ions than using EI [38]. But similar hindrances as for CEI apply to the instrumental setup of GC‐CI‐MS. Usually, a manual exchange of the ion source and flushing the analyzer chamber with reagent gas is incompatible with the process of routine analysis. Other approaches, like the application of an ESI source, are also not promising due to low tendency for ionization. This is supported by preliminary data as well as by findings from other studies [39, 40]. Therefore, ESI–MS is no practical option for THMT and 20OHnorTHMT.

## 5 Conclusions

For the two examples selected for this study, THMT and 20OHnorTHMT, each group exhibits a large diversity in diastereomers relevant for bioanalytical questions. These two groups of analytes do not even represent isomers nor isobars toward each other. Nevertheless, standard approaches by EI‐QTOF‐MS and EI‐QqQ‐MS are not able to distinguish among them reliably. Extensive fragmentation of both groups and a lack of detection of the molecular ion prevent identification by HRMS. Identical characteristic fragment ions *m/z* 255 and *m/z* 345 further hamper identification. In addition, shared ion transitions such as *m/z* 345.3 → 173.0 make MS/MS experiments not sufficient for discrimination among the two groups. Finally, co‐elution of **T5** and **N3** in a routinely applied GC method for anti‐doping analysis makes it clear that final identification is only possible with a well‐developed chromatographic separation. This was achieved by adjusting the already existing methods. The developed method with temperature program GC 3 provides the needed certainty for the assignment of the 16 targeted analytes. Finally, separation of **T5** + **N3** and **T1** + **T3** and no co‐elution among the groups of THMT nor 20OHnorTHMT was accomplished. Finally, this work improved existing approaches of detecting these metabolites of MD and MT and generally demonstrated the importance of chromatographic separation despite constant advances in mass spectrometry instruments. It was this extensive elaboration on the limitations of the mass spectrometric approach and the subsequent method development that enabled the application of this method in the analysis of an administration study of MD [22]. Furthermore, now achieving a clear identification of the analytes as a prerequisite the next step toward quantitation can be made.

## Author Contributions

**Jakob Steff**: methodology, investigation, formal analysis, validation, visualization, writing – original draft preparation. **Xavier de la Torre**: data curation, investigation, writing – review and editing. **Francesco Botrè**: resources, data curation, funding acquisition, writing – review and editing. **Maria Kristina Parr**: conceptualization, funding acquisition, project administration, supervision, methodology, resources, writing – review and editing.

## Supporting information




**Supporting File**: jssc70334‐sup‐0001‐SuppMat.docx.

## Data Availability

Data will be made available on request.
