# Supplementary material for: Overcoming the Challenge of Confident Identification Among Two Related Groups of 17‐Methyl Steroids by GC–MS
Source: J Sep Sci. 2025 Dec 18;48(12):e70334. doi: 10.1002/jssc.70334 (PMC12712887; doi:10.1002/jssc.70334)
Supplement: Supplementary file 1 — Supporting File: jssc70334‐sup‐0001‐SuppMat.docx. [file JSSC-48-e70334-s001.docx]

# Supplemental methods

## GC-MS

Method development of the chromatographic method was performed on a 7890 gas chromatograph (Agilent Technologies Inc., Santa Clara, CA, USA) coupled to a 5975C single quadrupole mass‑selective detector (Agilent Technologies Inc.) with helium as carrier gas with a constant flow rate of 1 mL/min. The inlet temperature was set to 300 °C, injection volume was 2 µL and split ratio 16:1. Ionization energy of 70 eV was applied and full scan mode ranged from *m/z* 40 to 1000.

In the following tested temperature programs depicted in Figure 2 are described.

### Method A – temperature program GC 2

The gas chromatograph was equipped with an Agilent HP1 column (17 m, 0.20 mm, 0.11 µm). The oven program was applied as indicated in Figure 2A: 183 °C, +3 °C/min to 232 °C, +40 °C/min to 310 °C, hold for 2 min.

### Method B

The gas chromatograph was equipped with an Agilent HP1 column (17 m, 0.20 mm, 0.11 µm). The oven program was applied as indicated in Figure 2B: 150 °C, +50 °C/min to 200 °C, +1.5 °C/min to 220 °C, hold for 2 min, +50 °C/min to 310, hold for 3 min.

### Method C

The gas chromatograph was equipped with an Agilent DB-5ms column (30 m, 0.25 mm, 0.25 µm). The oven program was applied as indicated in Figure 2C: 150 °C, +50 °C/min to 235 °C, +2 °C/min to 260 °C, hold for 6 min, +40 °C/min to 320, hold for 2 min.

### Method D – temperature program GC 3

The gas chromatograph was equipped with an Agilent DB-5ms column (30 m, 0.25 mm, 0.25 µm). The oven program was applied as indicated in Figure 2D: 150 °C, +50 °C/min to 220 °C, hold for 2 min, +5 °C/min to 235 °C, hold for 5 min, +2 °C/min to 245, hold for 4 min, +5 °C/min to 255 °C, +40 °C/min to 320, hold for 1 min.
